# Advances in the molecular pathogenesis of *Nocardia keratitis*

**DOI:** 10.3389/fcimb.2026.1861657

**Published:** 2026-07-22

**Authors:** Zheng-Wei Sun, Yi-Ming Guo, Lu Ye

**Affiliations:** 1 School of Medicine, Northwest University, Xi’an, Shaanxi, China; 2 Xi’an People’s Hospital (Xi’an Fourth Hospital), Affiliated People’s Hospital of Northwest University, Xi’an, Shaanxi, China

**Keywords:** *Nocardia keratitis*, host immunity, host-pathogen interaction, molecular mechanisms, PANoptosis, virulence factors

## Abstract

Nocardia keratitis is an infectious ocular disease that poses substantial diagnostic and therapeutic challenges worldwide. It occurs predominantly in individuals with corneal barrier disruption, contact lens wear, or impaired host immunity. Due to its relatively insidious onset, the clinical manifestations are nonspecific, and laboratory diagnosis is technically demanding. As a result, delayed identification and suboptimal treatment are common, often resulting in poor visual outcomes. Although diagnostic and therapeutic techniques have improved in recent years, a comprehensive understanding of Nocardia virulence mechanisms and their complex, dynamic interactions with the host immune system remains to be fully established. Therefore, starting from the temporal sequence of infection and the dynamic host-pathogen interplay, we integrate scattered observations into a pathological framework that traces the transition from colonization to immune dysregulation and ultimately to tissue damage. This review systematically outlines the major virulence factors of Nocardia implicated in the stages of adhesion and colonization, tissue invasion, and intracellular survival. It also discusses the dual role of neutrophils, emerging evidence for pyroptosis, apoptosis, and necroptosis (PANoptosis)-related signaling pathways, and the impact of host immune status on disease susceptibility. This work aims to provide a mechanistic basis for investigating the pathogenesis of Nocardia keratitis, optimizing its diagnosis, and developing targeted therapeutic strategies.

## 1 Introduction

The genus Nocardia comprises aerobic, Gram-positive, filamentous actinomycetes that are widely distributed in soil and aquatic environments and can act as opportunistic pathogens, causing a broad spectrum of infectious diseases, including ocular disease (Ong et al., 2023). More than 100 validly named Nocardia species have been identified, of which approximately 25 have been implicated in human ocular infections. Among corneal clinical isolates, *Nocardia cyriacigeorgica*, *Nocardia brasiliensis*, and *Nocardia asteroides* are most frequently encountered in clinical practice (Lalitha et al., 2007; Agarwal et al., 2021). The incidence of ocular infection shows marked geographic and sex-related variation. In temperate regions, Nocardia keratitis accounts for approximately 1–2% of bacterial keratitis cases, whereas in tropical and subtropical regions the proportion rises to 3–8%, with particularly high rates among agricultural workers in South and Southeast Asia (Lalitha, 2009). Eye contact with soil or plant matter is the most common precipitating factor, accounting for 48% of cases of Nocardia keratitis (DeCroos et al., 2011). Men exhibit a higher risk of infection, which has been attributed to increased environmental exposure and the lack of 17β-estradiol-mediated inhibition of intracellular pathogen survival (Han et al., 2022).

The clinical diagnosis and treatment of Nocardia keratitis have long faced multiple challenges. Its early manifestations have a certain similarity with Acanthamoeba keratitis, fungal keratitis, atypical mycobacterial keratitis, and other diseases. Conventional pathogen culture is time-consuming and often leads to the delay of treatment timing (Meera et al., 2021). In addition, Nocardia has limited susceptibility to some commonly used empirical antibacterial agents, which further increases treatment difficulty (Bartimote et al., 2019). More importantly, different Nocardia isolates show significant heterogeneity in drug resistance phenotypes, invasive capacity, and clinical severity (Adre et al., 2022; Guo et al., 2024), suggesting that reliance on species-level understanding alone is insufficient to explain its complex pathogenic spectrum. Genomic studies have revealed that the Nocardia genus has significant genomic plasticity. Among these, non-core genes such as genomic plasticity regions (RGPs) account for a large proportion of the genome, indicating that this genus can rapidly acquire exogenous genes through frequent horizontal gene transfer (Komaki et al., 2014). In contrast, conserved genes such as *sodA*, *pks13* and *mce* gene families constitute the basic pathogenic framework shared by almost all ocular pathogenic strains. These genes mediate antioxidant defense, mycolic acid synthesis, and host-cell invasion, respectively, and are the molecular basis for the establishment of ocular infection (Cai et al., 2022). This genomic perspective provides insight into the pathogenic differences among strains and also supports the development of specific diagnostic markers and the screening of anti-virulence targets for Nocardia keratitis.

Over the past decade, advances in genomics and functional studies have progressively elucidated Nocardia-associated virulence factors and the host immune regulatory pathways they engage. However, most existing work has focused on systemic nocardiosis, and the specific pathogenic features of Nocardia within the relatively immune-privileged corneal microenvironment have not been systematically characterized. Moreover, studies on virulence factors and host immune responses are largely fragmented, and an integrative analytical framework linking the temporal sequence of bacterial virulence programs with dynamic host immune responses during infection is still lacking. Against this backdrop, the key unresolved issue at present is how the bacterial pathogenic program interacts with a dysregulated corneal immune response, thereby driving the pathological process from a phase of microbial control toward tissue-destructive inflammation. This transition represents the critical turning point determining clinical outcomes. Therefore, this review focuses on the pathogenic course of Nocardia keratitis and the associated host–pathogen interactions, summarizes recent advances, highlights current controversies and knowledge gaps, and outlines potential directions for future research.

## 2 Temporal virulence programs in *Nocardia* corneal infection

### 2.1 Adhesion and colonization on the injured corneal epithelium

Epithelial defects caused by ocular trauma or contact lens wear are prerequisites for Nocardia adhesion and colonization, and represent the first rate-limiting step in the establishment of infection. Successful colonization of the cornea by Nocardia depends on a finely coordinated adhesion and retention system.

#### 2.1.1 Core molecular mechanisms of adhesion

Heparin-binding hemagglutinin (HBHA) is a highly conserved surface adhesin in ocular pathogenic Nocardia strains. It mediates the initial attachment of bacteria to host cells. Its lysine-rich C-terminal domain specifically binds to heparan sulfate and related glycosaminoglycan receptors, an interaction that has been confirmed in *in vitro* cultured cell lines. Additional adhesion-enhancing factors have been identified through genomic analysis and *in vitro* studies, which may act synergistically with HBHA to further enhance bacterial anchorage. For example, pili encoded by the homologous actinobacterial *flp* gene family mediate the specific binding between bacteria and host extracellular matrix proteins such as fibronectin. ϵ-poly-L-lysine produced by Nocardia neutralizes the acidic environment of phagosomes, thereby enhancing its intracellular survival capacity in macrophage models. Meanwhile, glucose metabolism-related proteins encoded by the *glc* operon help bacteria maintain adhesion and colonization in the nutrient-poor corneal stroma (Engelbrecht et al., 2021). However, it should be emphasized that the roles of these molecules in ocular models remain to be further validated. Collectively, these molecules constitute a complex regulatory network that plays an important role in stable colonization during the early stages of infection.

#### 2.1.2 Metal ion acquisition and support mechanisms

The extremely low concentrations of free iron and zinc in the cornea represent major barriers to early colonization by Nocardia. To overcome this limitation, Nocardia has evolved highly efficient metal ion uptake systems.

The highly conserved Nocobactin-type gene cluster forms the core of the iron acquisition system. The synthetase NbtS possesses a rare dual function: it is not only responsible for the biosynthesis of the siderophore nocobactin, which can effectively chelate free iron in the corneal microenvironment, but also functions as an independent virulence effector protein. It acts through the TLR4–MyD88–MAPK/NF-κB axis (Ji et al., 2022), thereby serving as a molecular hub linking bacterial metabolism to tissue damage. Its cytotoxic effects have been validated in studies of Nocardia pulmonary infection, but whether they exert the same effects in ophthalmic models remains to be directly confirmed. In addition, Nocardia can produce a variety of other siderophores, such as Nocactin and Transvalencin A, which exhibit potent iron-chelating activity and cytotoxicity in cell culture experiments (Engelbrecht et al., 2021). Analyses based on *in vitro* co-culture models of human neutrophils (PMN) and mouse macrophages have confirmed that heme operons (*hemB*, *hemE*, *hemH*, *hemN*) encoding enzymes involved in heme biosynthesis are present in nearly all ocular isolates, indicating that these operons may play a role in enhancing iron acquisition in the iron-deficient stromal microenvironment. These enzymes are also predicted to modulate the oxidative stress response, which may in turn enhance bacterial resistance to reactive oxygen species (ROS) produced by neutrophils (Lalitha, 2009). In the mouse macrophage co-culture system, Nocardia has been found to express Uniformide A, a novel zincophore, under zinc-limiting conditions that mimic the host tissue environment, which is presumed to facilitate persistent bacterial colonization in zinc-restricted niches (Hara et al., 2022).

### 2.2 Epithelial barrier disruption and corneal invasion

#### 2.2.1 Mammalian cell entry gene family

The mammalian cell entry (*mce*) gene family was first identified in *Mycobacterium tuberculosis* and encodes transmembrane proteins that mediate bacterial entry into mammalian cells. This gene family represents one of the best-characterized virulence determinants in Nocardia biology to date (Ji et al., 2017). Ophthalmopathogenic Nocardia strains typically harbor multiple *mce* gene clusters. Each gene cluster has been proposed to perform a distinct functional role at different stages of corneal infection. However, current research on *mce* gene clusters has been largely restricted to the functional validation of individual genes *in vitro*, and the mechanisms underlying the coordinated regulation of multiple *mce* gene clusters during corneal infection remain poorly understood. Furthermore, *in vivo* evidence from animal models remains extremely limited and is insufficient to confirm whether individual *mce* gene clusters perform distinct functions within the corneal microenvironment. *In vitro* functional studies suggest that these gene clusters may exhibit stage-specific roles during the progression of corneal infection: *mce1* mediates the initial disruption of the epithelial barrier; *mce5* facilitates early adaptation to the corneal microenvironment; *mce4* drives deep stromal invasion and intracellular survival; and *mce3* mediates uncontrolled inflammation and severe tissue necrosis. The functional specificities of different *mce* gene clusters as proposed on the basis of *in vitro* evidence are summarized in **Table 1**.

**Table 1:** Proposed functional roles and potential clinical associations of the nocardia *mce* gene family.

Gene Cluster	Potential Biomarkers	Molecular mechanisms proposed on the basis of *in vitro* studies	Proposed stages of involvement	Potential clinical associations and prognostic value	Type of Supporting Evidence	References
*mce1* (Core invasion operon)	Mce1C, Mce1D, Mce1E	Mce1C/D bind integrins and glycosaminoglycans on the surface of corneal epithelial cells, mediating bacterial internalization; they regulate host actin cytoskeletal rearrangement, facilitating entry into the cytoplasm; Mce1E promotes Th1-type adaptive immune activation by upregulating IFN-γ	Early epithelial barrier breach and initial invasion	Mce1C/D are factors in the early stages of colonization *in vitro*; an *in vitro* human corneal epithelial cell line model has shown that knocking out *mce1C/D* reduces bacterial invasion of corneal epithelial cells by more than 90%; the co-expression of Mce1C/D may serve as a potential marker for early disease progression.	*in vitro* functional assays of human corneal epithelial cell lines	(Ji et al., 2017)
*mce4* (Metabolic adaptation and intracellular survival operons)	Mce4C, Mce4F	Mediates cholesterol uptake and utilization, enabling bacteria to use cholesterol as an energy source in nutrient-deprived environments; facilitates bacterial entry into phagosomes containing TACO proteins, thereby preventing lysosomal degradation; enhances adaptation to the hypoxic corneal microenvironment	Stromal invasion and persistent intracellular infection in the middle and late stages	Genomic association finding: The *mce4F* gene is widely present in virulent strains from clinical Nocardia keratitis isolates. Functional inference: Targeted knockout of *mce4F* reduces bacterial virulence by more than 60% in *in vitro* and systemic infection models; sustained Mce4F expression may be associated with elevated chronic infection and recurrence risk; co-expression of Mce3C and Mce4F is a hypothetical biomarker for corneal stromal perforation and surgical intervention.	Genomic analysis of clinical ocular isolates; *in vitro* functional assays of macrophages and *in vivo* functional assays in pulmonary infection	(Yang et al., 2025)
*mce3* (Severe infection operon)	Mce3C	Activates the β2-integrin pathway to invade macrophages; inhibits phagosome-lysosome fusion and host-cell apoptosis; drives uncontrolled inflammatory responses by upregulating TNF-α and IL-6 and suppressing the anti-inflammatory factor IL-10	Late-stage severe infection and tissue necrosis	Preliminary studies have shown that Mce3C has been detected in isolates from patients with severe Nocardia keratitis; Mce3C positivity may serve as a candidate biomarker for disease severity and poor prognosis, but this requires validation in larger clinical cohorts	*In vitro* macrophage functional assays; preliminary observational correlation analysis of clinical isolates	(Yang et al., 2025)
*mce5* (Hypoxia adaptation operon)	Mce5A, Mce5E	Regulates lipid metabolism and enhances bacterial adaptation to the hypoxic microenvironment after infection; mediates weak adhesion to corneal epithelial cells	Early colonization support	Mce5 is primarily observed in isolates from patients with mild or self-limiting infections; its presence may be associated with low virulence and a favorable clinical outcome	Genomic analysis and preliminary observational correlation analysis of clinical ocular isolates	(Yang et al., 2025)

#### 2.2.2 Other invasion-related effector molecules

Beyond the *mce* gene family, hemolysins encoded by the *hly* operon form membrane pores and directly lyse host cells. Invasion-related genes such as *ibe* enhance bacterial entry into corneal endothelial and ciliary epithelial cells, enabling penetration of Descemet’s membrane and the blood-ocular barrier and increasing the risk of Nocardia endophthalmitis. In parallel, repeats in toxin (RTX) transport genes *rtxB* and *rtxE*, together with the endotoxin-related gene *pgi*, are widely distributed among Nocardia strains and cause time-dependent disruption of host cell membrane integrity. This pattern is consistent with the rapidly progressive tissue necrosis observed clinically in severe infections (Han et al., 2020). Secreted extracellular lipases and proteases from Nocardia degrade extracellular matrix components and collagen fibers, eliminating physical barriers to deep tissue invasion (Zoropogui et al., 2013).

### 2.3 Intracellular parasitism and establishment of a persistent state

Nocardia is a typical facultative intracellular pathogen capable of long-term survival and replication within corneal stromal cells, macrophages, and neutrophils, while employing multiple strategies to evade immune clearance. Intracellular parasitism underlies the chronic, relapsing clinical course and therapeutic refractoriness of Nocardia keratitis, and constitutes a mechanism of phenotypic resistance to first-line agents such as sulfonamides (Pawełczyk and Kremer, 2014).

Upon entry into host cells, Nocardia rapidly initiates intracellular survival programs. Effectors encoded by *ptpA* and *ndk* inhibit phagosomal acidification and phagosome-lysosome fusion, while hemolysins encoded by *hly* disrupt phagosomal membranes, releasing bacteria into the cytosol and thereby protecting them from hydrolytic enzymes (Yang et al., 2025). The *icl* gene encodes isocitrate lyase, which mediates the glyoxylate shunt. This allows bacteria to utilize host lipids as a carbon source and sustain survival in nutrient-deprived intracellular niches.

Under oxidative stress and nutrient limitation in the inflammatory microenvironment, Nocardia synthesizes guanosine tetraphosphate (ppGpp) via RelA, inducing a low-metabolic “persister” state (Nathar et al., 2024). This intracellular parasitism and persistence mechanism has been confirmed in macrophage models and systemic infection models. It enables Nocardia to evade both antimicrobial therapy and host immune attack, thereby establishing a long-term infectious reservoir within the cornea. This is considered one of the potential mechanisms underlying clinical relapse after apparent microbiological cure.

## 3 Multilevel immune evasion strategies of nocardia

The multilevel immune evasion strategies of Nocardia are proposed to be effective in the corneal microenvironment, primarily because of the intrinsic immune-privileged nature of this tissue. To preserve optical transparency and minimize inflammation-induced scarring, the cornea is characterized by sparse vascular and lymphatic networks and restricted immune cell infiltration. In addition, it exhibits a relatively high activation threshold for innate immunity, delayed recruitment of effector cells, and locally attenuated complement and adaptive immune responses. These features create a favorable environment for Nocardia’s low-inflammatory persistence, antioxidant defense, and immunosuppressive strategies, thereby facilitating the establishment of persistent, occult, and even recurrent infection on the ocular surface and within the stroma (Dempsey and Conrady, 2023).

### 3.1 Cell wall remodeling–mediated masking of immune recognition

Remodeling of cell wall components constitutes the first line of immune evasion in Nocardia, primarily mediated by mycolic acids and their derived glycolipids. Mycolic acids, synthesized by the *pks13* gene cluster (Burkovski, 2018), are major constituents of the mycolic acid-rich outer membrane and mask pathogen-associated molecular patterns (PAMPs), thereby reducing recognition by host immune receptors and dampening innate immune activation at its source. Trehalose monomycolate (TMM) and trehalose dimycolate (TDM), formed by mycolic acids and trehalose, act as important ligands for the C-type lectin receptor Mincle and TLR4, modulating the activation threshold of innate immunity and helping the pathogen establish a state of low-grade inflammatory persistence (Nishiuchi et al., 1999).

### 3.2 Evasion of phagocyte-mediated oxidative killing

Once within host tissues, resisting the oxidative burst of phagocytes is essential for Nocardia survival. Early animal studies demonstrated that administration of antibodies targeting Nocardia superoxide dismutase significantly reduces bacterial survival *in vivo*, underscoring the critical role of antioxidant enzyme systems in pathogenesis (Sridhar et al., 2001).

Nocardia has developed a coordinated antioxidant network centered on manganese superoxide dismutase (SodA), alkyl hydroperoxide reductase (AhpC), and catalase (KatA) (Wu et al., 2006). SodA not only eliminates extracellular superoxide anions and maintains intracellular ROS homeostasis (García-Hernández et al., 2014), but also promotes biofilm maturation and the synthesis of secondary metabolites, creating a positive feedback loop between antioxidant stability and enhanced virulence. Notably, in the early phase of macrophage infection, Nocardia may actively downregulate *sodA* transcription to evade early immune recognition (Revol et al., 2006). AhpC is highly conserved in ocular isolates and shows the highest expression abundance among virulence-associated genes, reflecting the deep coupling between antioxidant defense and metabolic stress-response systems (Nathar et al., 2024). This antioxidant system not only helps Nocardia resist phagocyte-mediated killing, but also provides a foundation for subsequent systemic immune suppression and chronic infection.

### 3.3 Systemic immune suppression and complement evasion

After overcoming immune recognition and intracellular killing, Nocardia constructs an immune evasion network spanning innate immunity, adaptive immunity, and the complement system, which is a critical mechanism for long-term infection and chronicity. The complement system is a core defense pathway at the ocular surface. Nocardia reduces C3b deposition on its surface through cell wall lipoarabinomannan, thereby lowering opsonophagocytic efficiency by macrophages. At the adaptive level, Nocardia establishes a multifaceted T-cell suppression network. Metabolites such as Brasilicardin A have been shown to inhibit T-cell activation (Arai et al., 2021), while infection-induced overactivation of the PD-1/PD-L1 axis has been shown to drive effector T-cell exhaustion in a murine actinomycetoma model. Additionally, bacterially driven NO production further suppresses T-cell proliferation, together markedly impairing T-cell–mediated protective immunity (Meester et al., 2013).

Inherited defects in the host immune system can markedly amplify these immune evasion effects. For example, CARD9 mutations impair C-type lectin receptor signaling, reduce Th17 activation and pro-inflammatory cytokine production, and weaken neutrophil bactericidal activity (Ali et al., 2020). Patients with chronic granulomatous disease(CGD), due to NADPH oxidase deficiency and complete loss of oxidative killing capacity, are highly susceptible to Nocardia infection (Martínez-Barricarte, 2020).

## 4 Host–pathogen interactions and mechanisms of corneal tissue damage

As an opportunistic pathogen, Nocardia relies on its virulence factors to initiate disease. However, a large body of evidence indicates that irreversible corneal damage is mainly driven by excessive activation of the host immune system in the context of incomplete pathogen clearance, with host–pathogen interactions driving disease progression (Lakhundi et al., 2017; Yetmar et al., 2025).

### 4.1 Corneal barrier disruption and initial immune recognition

Under physiological conditions, the physical and chemical barriers of the cornea restrict direct contact between pathogens and the immune system. The corneal surface is covered with microvilli, glycocalyx, and a tear film; the tear film contains antimicrobial factors such as lysozyme, lactoferrin, and lipocalin, which inhibit bacterial adhesion and sequester iron ions (O'Callaghan et al., 2003). In addition, resting corneal epithelium exhibits low basal expression levels of TLR2 and TLR4, as well as intrinsic hyporesponsiveness to TLR4 ligands such as lipopolysaccharide (LPS). This hyporesponsiveness prevents inappropriate inflammatory responses to commensal bacteria or environmental microorganisms.

This delicate balance is rapidly disrupted once the integrity of the corneal barrier is compromised. Pattern recognition receptors (PRRs) on the surface of epithelial cells, resident immune cells, and early infiltrating leukocytes recognize Nocardia-derived PAMPs. This recognition activates both MyD88-dependent and MyD88-independent signaling pathways, which collectively trigger the MAPK and NF-κB cascades. These signaling events drive the recruitment of neutrophils and monocytes to the site of infection and mediate the host’s initial immune response against Nocardia (Ji et al., 2020). Notably, although infection induces early upregulation of both TLR2 and TLR4 expression, these two receptors exhibit distinct temporal expression patterns: TLR2 expression remains elevated throughout the entire course of infection, whereas TLR4 expression in mast cells is only transiently upregulated in the early post-infection period and then undergoes gradual and sustained downregulation (Millán-Chiu et al., 2011). This selective downregulation of TLR4 is considered an immune evasion strategy employed by Nocardia, which impairs the host’s ability to clear the pathogen and promotes the establishment of chronic infection. The key PRRs involved and their functional characteristics are summarized in **Table 2**.

**Table 2:** Pattern recognition receptors involved in immune recognition in nocardia keratitis.

Receptor	Recognized ligand	Tissue localization and dynamic expression characteristics	Functional roles and dynamic regulation	Type of Supporting Evidence	References
TLR2	Polysaccharides, lipoproteins, lipoteichoic acid	Resting corneal epithelium exhibits low basal expression; expression is significantly upregulated in neutrophils, macrophages and corneal epithelial cells during early infection, and remains elevated throughout the entire course of infection	Drives early pro-inflammatory responses and neutrophil recruitment; sustained activation during chronic infection exacerbates stromal inflammatory damage and may impair the antigen-presenting function of macrophages, thereby promoting persistent pathogen colonization.	Validated in studies of chronic granuloma models and subcutaneous soft tissue infection models, as well as in cultured macrophage cell lines *in vitro*	(Millán-Chiu et al., 2011; Ji et al., 2020)
TLR4	Mycolic acids, HBHA	Under physiological conditions, TLR4 expression in the corneal epithelium is extremely low and localizes exclusively to intracellular vesicles, with no expression on the cell membrane surface, resulting in intrinsic functional unresponsiveness to ligands such as LPS. Following corneal barrier disruption, intracellular TLR4 rapidly translocates to the cell membrane and undergoes rapid upregulation in corneal epithelial cells, macrophages and mast cells. However, this upregulation is only transient in mast cells and is followed by gradual and sustained downregulation during the mid-to-late stages of infection.	Restores functional recognition of Nocardia-derived PAMPs after barrier disruption, initiating the early innate immune response and macrophage activation; selective downregulation of TLR4 in mast cells at later stages impairs the host’s ability to clear the pathogen and promotes the establishment of chronic infection	Validated in *in vitro* macrophage infection models and animal studies of nocardial mycetoma	(Millán-Chiu et al., 2011; Pandey et al., 2013)
TLR5	Flagellin	Under resting conditions, it is expressed exclusively in the basal and wing cell layers of the corneal epithelium; following disruption of epithelial barrier integrity, it is activated and its expression is upregulated.	Specifically recognizes flagellin from motile Nocardia strains, regulates innate immune responses and mediates neutrophil recruitment to corneal infection foci	Deduced from studies on mycobacterial keratitis; no direct functional verification has been performed in Nocardia keratitis models	(Pandey et al., 2013)
TLR9	Unmethylated CpG DNA	Low basal expression in corneal epithelial cells and resident immune cells under resting conditions; significantly upregulated after infection and specifically localizes to endosomal membranes	Recognizes intracellular Nocardia DNA, induces secretion of type I interferons and pro-inflammatory cytokines, and enhances immune responses against intracellular pathogens	Deduced from studies on mycobacterial keratitis; no specific verification data for Nocardia keratitis are available	(Connell, 2001)
Mincle (CLR)	Mycolic acid-associated glycolipids	Mainly expressed on the surface of macrophages, dendritic cells and neutrophils; significantly upregulated after infection, co-localizes and acts synergistically with TLR2	Synergizes with TLR2 to recognize Nocardia cell wall glycolipid components, amplifies pro-inflammatory signals, and drives the release of inflammatory cytokines and chemokines; participates in the regulation of chronic granuloma formation and persistent pathogen infection	Deduced from systemic Nocardia infections; simultaneously validated in cultured macrophage cell lines *in vitro*	(Sridhar et al., 2001)

### 4.2 Direct cell and tissue injury mediated by virulence factors

After early defenses are breached, tissue and cellular damage in Nocardia keratitis is mediated by virulence factors. The Nocardia genome is enriched in polyketide synthase (PKS) and non-ribosomal peptide synthetase (NRPS) gene clusters, which encode multiple cytotoxic secondary metabolites in response to host microenvironmental cues (Connell, 2001). Among these, the polyketide nargenicin has been shown to induce apoptosis in cultured human corneal stromal and epithelial cell lines and suppress VEGF/VEGFR2 signaling *in vitro*, thereby impairing angiogenesis and corneal repair processes (Engelbrecht et al., 2021). Benzoanthraquinones and alkaloid metabolites exhibit strong cytotoxicity and can directly damage cell membranes, exacerbating ulceration and necrosis (Herisse et al., 2020). In addition, Nocardia secretes multiple proteases and lipases that degrade extracellular matrix and collagen fibers, providing structural support for deep tissue invasion. Beyond direct cytotoxicity, these metabolites can interfere with Wnt signaling and proteasomal function, suppressing corneal repair and immune activation (Barry and Beaman, 2007). At this stage, direct bacterial cytotoxicity begins to overlap with immune-mediated injury, further aggravating disease progression.

### 4.3 Indirect tissue injury driven by excessive host inflammatory responses

Following PRR recognition and stimulation by virulence factors, MAPK, NF-κB, and inflammasome pathways together form a complex and highly coordinated signaling network.

The MAPK subpathways have distinct roles in inflammatory regulation: JNK is required for HBHA-induced TNF-α expression; p38 promotes inducible nitric oxide synthase expression, forming a pro-inflammatory positive feedback loop; and ERK and JNK cooperate in regulating IL-6 and the immunoregulatory cytokine IL-10. Different Nocardia strains vary substantially in their ability to activate MAPK signaling, a difference that contributes to heterogeneity in inflammatory severity and clinical phenotype among patients (Assabayev et al., 2024).

The NF−κB pathway represents a central hub in the inflammatory cascade. In the canonical activation mode, engagement of pattern-recognition receptors leads to MyD88−dependent signaling, phosphorylation and subsequent degradation of IκBα, and the release of NF−κB dimers such as p65/p50, which then translocate into the nucleus and initiate transcription of downstream target genes. Concurrently, activated NF-κB signaling drives robust recruitment of neutrophils to the corneal lesions, amplifies the matrix metalloproteinase cascade, and ultimately leads to irreversible visual impairment (Das et al., 2022). Notably, several virulence factors of Nocardia spp., such as Uniformide B, can stabilize IκBα or interfere with p65 phosphorylation and nuclear translocation, leading to suppression of NF−κB activity, downregulation of pro−inflammatory cytokine production, and impairment of macrophage function, which together facilitate immune evasion and the establishment of chronic colonization by the pathogen.

In addition, both NLRP3 and NLRC4 inflammasomes are activated during the pathogenesis. Through caspase-1 activation, they mediate the maturation of IL-1β and IL-18, further amplifying the inflammatory response. Animal studies in other bacterial keratitis models have shown that inhibition of NLRP3 inflammasome activation significantly reduces corneal inflammation and tissue damage, supporting the plausibility of its involvement in disease progression (Taube et al., 2015). With sustained activation of these signaling pathways, the local immune response shifts toward a pathological pattern characterized by amplified inflammation and tissue damage. Accordingly, corneal tissue damage results from both bacterial cytotoxicity and the evolution of the host response, and this is also reflected in neutrophil biology.

### 4.4 Neutrophil-mediated bidirectional effects and tissue destruction

Driven by the inflammatory cascades described above, neutrophils act as central effector cells of the innate immune response and simultaneously as major drivers of irreversible corneal tissue injury, displaying a pronounced dual role in pathogenesis. Studies have shown that during early infection, neutrophils clear pathogens through phagocytosis, respiratory burst, and neutrophil extracellular trap (NET) formation. In later stages, sustained and excessive neutrophil infiltration leads to the release of large amounts of ROS, elastases, and matrix metalloproteinases (MMPs), driving irreversible stromal degradation and lesion progression (Nishida et al., 2021). MMP-9, in particular, can cleave and activate precursor IL-1β, further intensifying inflammation and establishing a vicious cycle of “neutrophil infiltration–MMP release–tissue damage–additional neutrophil recruitment.” Clinical studies have confirmed that doxycycline, as an MMP inhibitor, can alleviate corneal melting, providing direct clinical validation for this mechanism (García-López et al., 2023). In addition, the IL-1β/IL-1R1 axis plays a critical role in regulating immune cell infiltration. Studies have shown that IL-17 produced by γδ T cells drives early neutrophil migration in systemic Nocardia infection models; inhibition of IL-17 function leads to a marked increase in bacterial load (Leal and Pearlman, 2012; Tam et al., 2012). This dual effect of neutrophils makes their dynamic molecular profile a potential biomarker for diagnosis and prognosis.

### 4.5 Programmed cell death and PANoptosis

**Figure 1:**
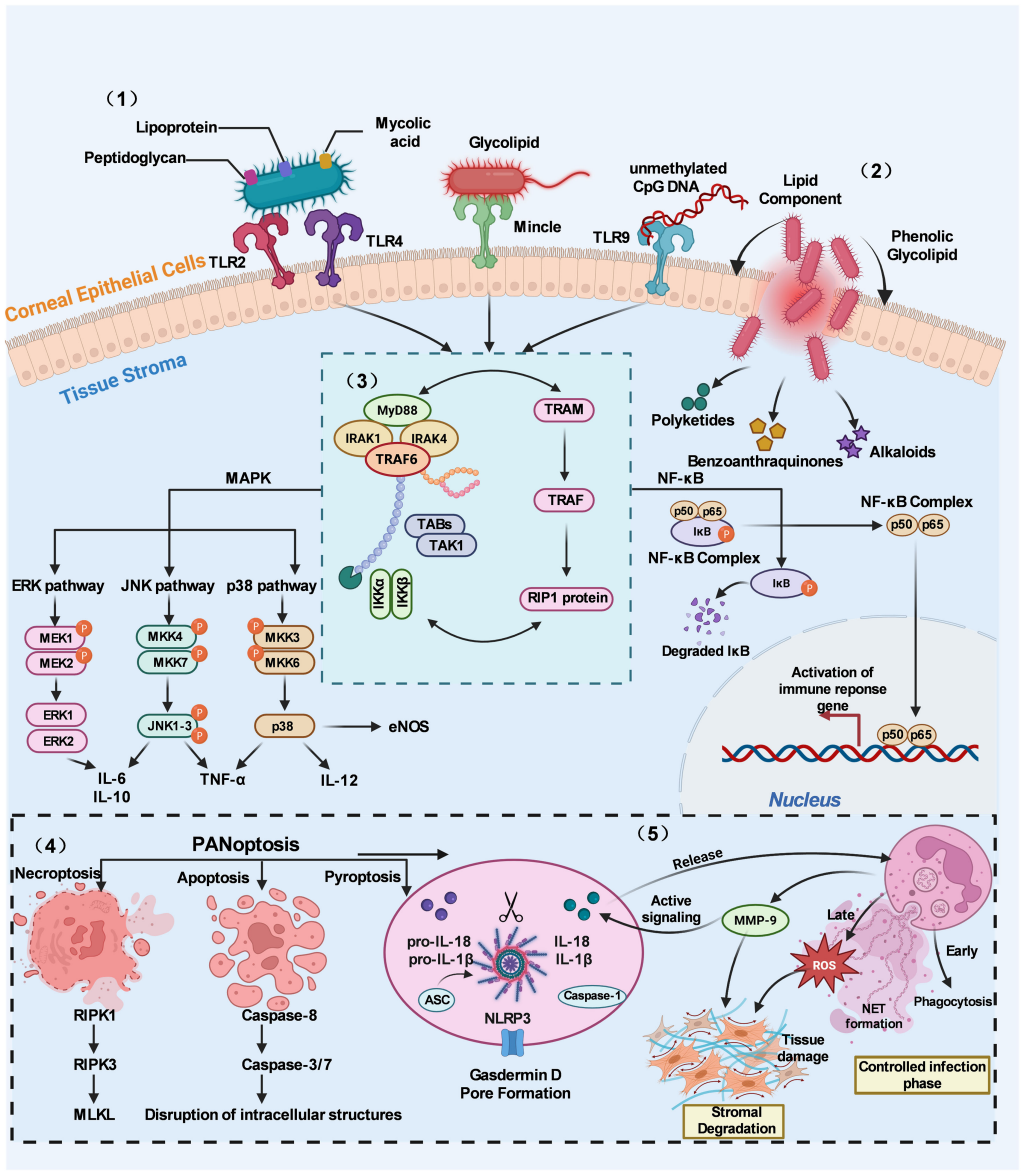
Host-pathogen interactions and molecular pathogenesis in Nocardia keratitis. This schematic diagram illustrates the intracellular molecular cascade of host-pathogen interactions in Nocardia keratitis (Ong et al., 2023). Toll-like receptors TLR2, TLR4, TLR9 and the C-type lectin receptor Mincle expressed on corneal epithelial and immune cells recognize Nocardia-derived pathogen-associated molecular patterns (PAMPs), initiating innate immune activation (Agarwal et al., 2021). Secreted bacterial metabolites, including polyketides, benzoanthraquinones and alkaloids, further stimulate inflammatory signaling pathways (Lalitha et al., 2007). Activated receptors engage both MyD88-dependent and -independent pathways, converging on MAPK and NF-κB cascades. The MAPK pathway, comprising ERK, JNK and p38 modules, phosphorylates downstream targets and drives transcription of cytokines such as IL-6, IL-10, TNF-α and IL-1β. In parallel, NF-κB signaling promotes IκB phosphorylation and ubiquitin-dependent degradation, releasing p50/p65 dimers that translocate into the nucleus to induce pro-inflammatory gene expression (Lalitha, 2009). Persistent inflammatory activation engages the NLRP3 inflammasome and may trigger PANoptosis, an emerging mechanistic framework characterized by the simultaneous activation of apoptosis, pyroptosis, and necroptosis. If confirmed in Nocardia keratitis, it will help elucidate the mechanisms underlying corneal cell death and the maturation of inflammatory cytokines (DeCroos et al., 2011). Neutrophils exert stage-dependent effects: in early infection they contribute to pathogen clearance via phagocytosis and neutrophil extracellular trap (NET) formation, whereas in later stages excessive production of reactive oxygen species (ROS) and MMP-9-mediated stromal degradation leads to progressive tissue damage.

When the cumulative damage caused by inflammatory injury and pathogenic factors exceeds the repair capacity of corneal tissue, corneal cells may initiate programmed cell death. Evidence from studies of bacterial keratitis and systemic infection models indicates that this response is characterized by the simultaneous activation of pyroptosis, apoptosis, and necroptosis, collectively referred to as PANoptosis. Although direct evidence for a coordinated role of PANoptosis in Nocardia keratitis is currently lacking, PANoptosis has been proposed as an emerging framework for corneal cell death, ulcer formation, and stromal lysis in infectious keratitis. Among these pathways, pyroptosis mediated by the NLRP3 inflammasome–caspase-1–GSDMD axis is the most extensively studied. Activated caspase-1 cleaves GSDMD to form membrane pores, leading to cell lysis and the release of pro-inflammatory cytokines IL-1β and IL-18, thereby amplifying the inflammatory cascade and promoting ulcer progression. Meanwhile, Nocardia infection can induce host cell apoptosis through the caspase-8/caspase-3/7 pathway and necroptosis through the RIPK1–RIPK3–MLKL axis (Lin et al., 2022; Wang et al., 2025). Notably, in a *Nocardia brasiliensis*-induced subcutaneous lesion model, endothelial nitric oxide synthase (eNOS) was found to produce nitric oxide (NO) in infected tissues. It can both inhibit T cell proliferation and promote immune evasion, as well as induce apoptosis and amplify inflammation, thereby forming a vicious cycle of “NO production-T cell suppression-inflammation amplification-tissue destruction”, which ultimately leads to rebound of local bacterial load and chronic persistent infection (Salinas-Carmona et al., 2020; Vazquez-Marmolejo et al., 2021). However, this knockout phenotype has not been validated in the Nocardia keratitis model, and the specific contribution of the eNOS-NO axis to the pathogenesis of corneal infection remains to be definitively determined. Existing evidence suggests that pyroptosis, apoptosis, and necroptosis may not be isolated events in Nocardia-associated tissue damage, but rather occur in a parallel or cross-regulatory manner, resulting in tissue damage that is far more severe than that induced by any single cell death mode alone. The pathogenic cascade of Nocardia keratitis and a summary of the PANoptosis infection model are shown in **Figure 1**.

### 4.6 Host immune status and susceptibility to infection

The overall immune status of the host influences all of the above processes and ultimately determines the risk of Nocardia keratitis, disease severity, and treatment response. Protective immunity against Nocardia has long been thought to involve both cellular and humoral components, although their relative contributions remain incompletely defined. Th1-centered cellular immunity depends critically on intact signaling through the IL-12/IL-23–IFN-γ axis. IL-12 drives naïve T-cells to differentiate into Th1 effector cells, and IFN-γ produced by Th1 cells activates macrophages and enhances intracellular killing of pathogens. Any defect at any node of this axis can compromise host defense and predispose to severe infection. Clinical and genetic evidence supports this view: in patients with AIDS, a CD4^+^ T-cell count below 35/µL is associated with more than a 20-fold increase in the risk of Nocardia infection and an enucleation rate exceeding 70% (Pintado et al., 2003). Likewise, IL-12Rβ1 or IL-12p40 deficiency completely blocks IL-12/IL-23 signaling and markedly reduces IFN-γ production (Picard et al., 2002; Luangwedchakarn et al., 2009). Humoral immunity, centered on pathogen-specific antibody responses, also plays an indispensable complementary role in host defense (Pedraza et al., 2010). Studies have shown that Nocardia-specific IgM enhances recognition and clearance of extracellular bacteria by phagocytes through dual mechanisms of opsonization and complement activation, thereby functionally complementing Th1-type cellular immunity (Salinas-Carmona et al., 2006). However, it does not act on intracellular persister bacteria and therefore cannot replace the central role of cellular immunity. Taken together, current evidence indicates that protective immunity against Nocardia is best understood as a cooperative defense mechanism dominated by Th1-type cellular immunity with supportive contributions from humoral responses.

Prolonged local or systemic use of glucocorticoids is another major risk factor for Nocardia keratitis, and the controversy surrounding their clinical use ultimately stems from their suppressive effect on Th1-mediated protective immunity. Subgroup analysis of the Steroids for Corneal Ulcers Trial (SCUT) confirmed that topical glucocorticoid use during the active phase of infection suppresses Th1 function and worsens corneal infiltration and scarring (Lalitha et al., 2012; Srinivasan et al., 2014). By contrast, small retrospective studies suggest that low-dose glucocorticoids after infection control may reduce inflammation-induced stromal melting (Soleimani et al., 2020). Critical appraisal suggests that the heterogeneity of these findings reflects the combined effects of timing of administration, dosing regimen, and host immune status. Glucocorticoid use before adequate control of bacterial burden suppresses host protective immunity and is associated with a clear risk of clinical harm; high-dose or prolonged use increases the risk of severe infection and poor outcomes. In addition, adverse events are more likely to occur in immunocompromised patients.

## 5 Emerging therapeutic strategies for *Nocardia* keratitis

### 5.1 Anti-virulence targeted therapy

Based on the pathogenic mechanisms and host-mediated tissue injury described above, current therapeutic research is shifting from conventional antimicrobial therapy toward combined strategies incorporating anti-virulence agents, host-directed interventions, and precision drug delivery technologies. Anti-virulence therapy is conceptually attractive as it neutralizes pathogenic mechanisms without imposing selective pressure for antimicrobial resistance, making it particularly suitable for the early stages of infection where viable bacteria predominate. Monoclonal antibodies targeting HBHA or Mce1C/Mce1D have been proposed as anti-adhesive or anti-invasive strategies, but supporting evidence remains largely mechanistic or derived from non-corneal systems, and these approaches should currently be considered exploratory and hypothesis-generating research. Another promising target is the siderophore biosynthetic enzyme NbtS; its inhibition may simultaneously block bacterial iron acquisition and suppress TLR4-MyD88-NF-κB/MAPK-mediated inflammatory signaling (Shen et al., 2024). Similarly, inhibition of the *pks13* gene cluster disrupts mycolic acid biosynthesis, compromises cell wall integrity, and enhances immune clearance. Despite these encouraging advances, systematic analyses of these targets across clinically relevant pathogenic Nocardia species remain limited, and the marked strain heterogeneity within the genus suggests that single-target agents may not provide adequate coverage for all clinically important species.

### 5.2 Host-directed therapy

Host-directed therapy (HDT), as an emerging strategy for Nocardia keratitis, has attracted increasing attention. Its primary objective is to achieve a more precise balance between pathogen clearance and tissue protection by modulating host immune responses. During the early stages of infection, enhancing the antimicrobial functions of neutrophils and macrophages may facilitate pathogen clearance, although current evidence is primarily derived from *in vitro* studies and systemic infection models. In the late stages of the disease, strategies targeting excessive host inflammatory responses represent the main focus of current research. Preclinical studies in other bacterial keratitis models have demonstrated that targeted antagonism of TLR2/TLR4 pattern recognition receptors and their downstream MAPK and NF-κB inflammatory signaling pathways can block the amplification of inflammatory cascades mediated by pathogen invasion at the source. However, direct validation in Nocardia keratitis remains lacking. Similarly, blockade of the PD-1/PD-L1 axis has been proposed as a strategy to reverse T-cell exhaustion and promote pathogen clearance in persistent infection models, but no efficacy or safety data specific to the cornea are available (Vázquez-Marmolejo et al., 2025). Therefore, given the potential risk of exacerbating ocular inflammation, these strategies should be considered speculative and require careful evaluation. Looking ahead, the combination of HDT, rapid point-of-care diagnostics, and drug delivery systems holds promise for achieving a balance between pathogen clearance and inflammation control in the treatment of Nocardia keratitis, thereby improving clinical outcomes (Duarte-Mata and Salinas-Carmona, 2023).

### 5.3 Novel drug delivery systems

The clinical translation of both conventional antibiotics and the aforementioned emerging therapies is constrained by the intrinsic limitations of ocular topical delivery. These limitations include restricted drug penetration across the corneal epithelial barrier, difficulty maintaining therapeutic concentrations at the lesion site, and insufficient intracellular delivery efficiency. One approach involves the use of lipid-based or polymer-based nanoparticles and dendrimer carriers, which may enhance the delivery of antibiotics and anti-virulence agents to the corneal stroma and infected cells (Jaiswal et al., 2024). Drug-loaded contact lenses maintain therapeutic concentrations while enabling sustained local release and reducing dosing frequency, thereby improving patient adherence (Alamillo-Velazquez et al., 2021). Bioactive hydrogels are currently under development to achieve controlled, long-term release of growth-promoting factors and accelerate epithelial healing (Tsai et al., 2023; Yu et al., 2025). These innovative drug delivery technologies may help overcome the biological barriers of the eye and improve treatment efficacy in Nocardia keratitis. However, despite substantial progress, the translation from laboratory research to personalized clinical therapy still faces multiple challenges, including biocompatibility evaluation, long-term safety validation, and optimization of large-scale manufacturing processes, all of which require further clinical trials and broader implementation studies.

## 6 Discussion

Nocardia keratitis is a globally challenging blinding eye disease that remains difficult to diagnose and treat. Based on currently published research, we have proposed an immunological interaction model for Nocardia keratitis. In the early stage of infection, Nocardia adheres to the damaged corneal epithelium through pili, and putatively through HBHA and other molecules, and completes initial colonization with the assistance of its siderophore system. Subsequently, proteins encoded by the *mce* gene family drive epithelial penetration and deep stromal invasion, activating signaling pathways such as MAPK and NF-κB, which promote the release of pro-inflammatory cytokines and recruitment of neutrophils and thereby support host defense against the pathogen. Preliminary clinical studies suggest an association between the presence or absence of specific *mce* gene expression and disease prognosis; however, this requires further validation in larger sample sizes. As infection persists, inflammatory signaling becomes chronically overactivated, and the function of neutrophils gradually shifts from bactericidal defense to a major source of corneal stromal damage. This process is accompanied by inflammasome activation and the potential occurrence of PANoptosis. Although direct evidence for coordinated activation in Nocardia keratitis is currently lacking, if confirmed, PANoptosis could lead to more extensive corneal cell death and further aggravate injury. Notably, even when pathogen burden decreases or cultures become negative, sustained host inflammatory activation can continue to drive the progression of tissue injury independently. Meanwhile, Nocardia establishes a multilayered immune evasion network through cell wall remodeling, complement evasion, and suppression of adaptive immunity, effectively prolonging survival in the cornea and hindering pathogen clearance, which poses major challenges for clinical prevention and treatment.

From the perspective of disease progression, Nocardia keratitis is more appropriately defined as a “stage-switching” infection, rather than a simple superposition of single virulence factors. Different members of the *mce* gene family mediate distinct pathological processes, suggesting that Nocardia does not act through a constant pathogenic pattern. Instead, it dynamically adjusts its survival and virulence strategies in response to changes in the corneal microenvironment. Pathogenicity therefore depends not on the absolute potency of a single virulence molecule, but on whether the temporal virulence program can be executed completely and continuously in the corneal environment. Thus, the final severity of disease essentially reflects whether the bacteria can successfully complete the full pathophysiological sequence from surface colonization to deep stromal invasion and inflammatory amplification. The inherent characteristics of the cornea, including low vascularity and physiological immune suppression, provide uniquely favorable conditions for this sequential process. However, most existing studies rely on static observations at discrete time points and therefore make it difficult to define the spatiotemporal evolution of the signaling pathways that drive disease progression. Future studies should integrate single-cell omics and spatial transcriptomics to reveal the functional remodeling of key cell populations at different stages of disease.

A deeper understanding of the pathogenesis of Nocardia keratitis has important clinical implications. The continued progression of corneal ulcers after negative culture results suggests that late-stage tissue injury is no longer fully dependent on viable bacterial burden (Song et al., 2016), but may instead be driven by a self-sustaining inflammatory circuit composed of the MAPK/NF-κB axis, inflammasomes, and PANoptosis. At this stage, attributing all clinical deterioration simply to inadequate infection control and intensifying antibacterial treatment indiscriminately may increase the risk of ocular drug toxicity. Instead, the decisive turning point in disease progression lies in the irreversible transition of the host immune response. This also explains why immunosuppressive therapy exhibits obvious time-dependent biphasic effects. Premature intervention may weaken pathogen clearance and facilitate dissemination, whereas delayed intervention may fail to reverse already initiated stromal lysis. Therefore, future work should focus on cross-regulatory signaling networks and combine them with high-throughput drug screening to identify molecular switches and biomarkers that mark the transition from infection-dominant to damage-dominant disease, with gene editing and single-cell sequencing used for *in situ* functional validation of candidate targets.

Based on these pathophysiological characteristics, the main bottleneck in the clinical translation of Nocardia keratitis research is not a lack of potential therapeutic targets, but rather the inability to determine the optimal timing of intervention with precision. Existing evidence indicates that TLR signaling antagonism, PD-1/PD-L1 modulation and glucocorticoid therapy all exhibit stage-dependent biphasic effects (Sun et al., 2023). However, there are currently no validated clinical tools to determine dynamically whether the pathogen has been completely cleared or whether inflammation has shifted to host-driven sterile inflammation. The key translational gap lies in the lack of a risk-stratification system and rapid point-of-care diagnostic tools for Nocardia keratitis, making it difficult to distinguish persistent infection from sterile inflammation. Therefore, current clinical research should prioritize the development of a stratified assessment framework that can distinguish the pathogen-clearance phase from the inflammation-dominant phase by integrating dynamic pathogen monitoring, imaging features, and immune biomarkers (Austin et al., 2017).

In conclusion, this review systematically summarizes the virulence factors of Nocardia and their dynamic interactions with the host immune system across the stages from initial colonization to final tissue damage, analyzes the major bottlenecks in current clinical translation, and proposes directions for future research. We emphasize that the pathogenesis of Nocardia keratitis does not lie in the independent action of a single molecule, but in the dynamic coupling between the pathogen’s temporal virulence program and the host’s threshold-dependent immune imbalance. Recognizing this pathological trajectory shifts the conceptual focus from isolated virulence factors to the integrated pathophysiological process of the disease.
